# Total radical gastrectomy under continuous thoracic epidural anaesthesia

**DOI:** 10.4103/0019-5049.68385

**Published:** 2010

**Authors:** S Parthasarathy, M Ravishankar, U Aravindan

**Affiliations:** Department of Anaesthesiology, Govt. Dist Headquarters Hospital, Kumbakonam, Chennai, India; 1 Mahatma Gandhi Medical College and Research Institute, Pondicherry, India; 2 Department of GE Surgery, Thanjavur Medical College, Thanjavur, India

Sir,

We wish to report a case of radical gastrectomy, planned for combined general and epidural, but completed under continuous segmental thoracic epidural anaesthesia alone. A 72-year-old, 70-kg male who earlier underwent endoscopic biopsy, diagnosed as adenocarcinoma stomach, was posted for radical gastrectomy. In preoperative evaluation, the cardiorespiratory system was normal. His airway evaluation revealed a mallampatti class III and decreased atlanto occipital extension. The other airway parameters were within acceptable limits. He was a non-diabetic but a mild hypertensive, hence was on treatrment with amlodipine. His routine investigations including an X-ray of the chest, and ECG and heart echo were normal. The plan was to insert a thoracic epidural catheter and a central line before administering general anaesthesia. A central line was placed in the right internal jugular vein and an 18 G epidural catheter was inserted in the T5–T6 interspace. Routine difficult airway equipments were kept ready. After pre-oxygenation, the patient was induced with fentanyl 50 *μ*g, atropine 0.6 mg, thiopentone 250 mg and suxamethanium 120 mg. The larynx was not visualized after routine manipulations. After three unsuccessful attempts, a size 4 LMA was inserted to maintain airway and oxygenation. After 10 min, there were adequate respiratory attempts and the LMA was removed. In view of the easy and successful maintenance of airway with the LMA, it was decided to go ahead with epidural alone. The LMA could not be continued due to possibility of many manoeuvres with Ryle’s tube in the intra-operative period. Eight millilitres of 0.5% bupivacaine with 2 mg midazolam injected through the epidural catheter maintained satisfactory anaesthesia. After 100 min, another 8 ml of 0.5% bupivacaine with 50 *μ*g of fentanyl was injected into the epidural catheter. This was added to counter the problem of visceral pain. There was a mild discomfort with oesophageal manipulation which decreased with 50 *μ*g of IV fentanyl. The surgeon considered the relaxation to be adequate but felt that it could have been better. After another 45 min, the next supplementation with 8 ml of 0.5% bupivacaine through the epidural catheter was done. The surgery was completed in around 4.5 h. The intra-operative period was normal. The postoperative period was uneventful with analgesia through the epidural catheter. Radical gastrectomy for cancer of the stomach[1] is the treatment of choice in adequately prepared and diagnosed patients. Tomoko *et al*.,[2] in their study of combined epidural and general anaesthesia showed better postoperative pain relief with the combined technique than general anaesthesia alone. In our case, we planned the same but due to failed intubation we had to proceed with epidural alone. Our place is a peripheral centre with no additional gadgets like fibrescope for assisting airway management. Thoracic epidural[3] with a brachial plexus block has been used without general anaesthesia for radical mastectomy. It has been shown that thoracic epidural catheterization[4] for abdominal and thoraco-abdominal surgery is not associated with a high incidence of serious neurological complications. In fact, the incidence of puncture and catheter-related complications are less in the mid and upper than in lower thoracic region. Our patient did receive the catheter in the T5–T6 interspace. Sun[5] has demonstrated in his study that subtotal gastrectomy can be done with epidural anaesthesia alone without general anaesthesia. They have combined acupuncture to get better intra-operative results. But in our case it was radical gastrectomy which we could finish with minor hiccups. We conclude that continuous segmental thoracic epidural anaesthesia as a single technique is possible for major abdominal surgeries in patients with difficult airways and when situations force us to use such methods especially in peripheral set-ups. The addition of neuraxial opioids and midazolam might have contributed for an acceptable intra-operative course.
